# Antiarrhythmic Effects of Carvedilol and Flecainide in Cardiomyocytes Derived from Catecholaminergic Polymorphic Ventricular Tachycardia Patients

**DOI:** 10.1155/2018/9109503

**Published:** 2018-04-12

**Authors:** R. P. Pölönen, K. Penttinen, H. Swan, K. Aalto-Setälä

**Affiliations:** ^1^Faculty of Medicine and Life Sciences and BioMediTech Institute, University of Tampere, Tampere, Finland; ^2^Helsinki University Central Hospital, Helsinki, Finland; ^3^Heart Center, Tampere University Hospital, Tampere, Finland

## Abstract

Mutations in the cardiac ryanodine receptor (RYR2) are the leading cause for catecholaminergic polymorphic ventricular tachycardia (CPVT). In this study, we evaluated antiarrhythmic efficacy of carvedilol and flecainide in CPVT patient-specific induced pluripotent stem cell-derived cardiomyocytes (iPSC-CMs) carrying different mutations in RYR2. iPSC-CMs were generated from skin biopsies of CPVT patients carrying exon 3 deletion and L4115 or V4653F mutation in RYR2 and of a healthy individual. Ca^2+^ kinetics and drug effects were studied with Fluo-4 AM indicator. Carvedilol abolished Ca^2+^ abnormalities in 31% of L4115F, 36% of V4653F, and 46% of exon 3 deletion carrying CPVT cardiomyocytes and flecainide 33%, 30%, and 52%, respectively. Both drugs lowered the intracellular Ca^2+^ level and beating rate of the cardiomyocytes significantly. Moreover, flecainide caused abnormal Ca^2+^ transients in 61% of controls compared to 26% of those with carvedilol. Carvedilol and flecainide were equally effective in CPVT iPSC-CMs. However, flecainide induced arrhythmias in 61% of control cells. CPVT cardiomyocytes carrying the exon 3 deletion had the most severe Ca^2+^ abnormalities, but they had the best response to drug therapies. According to this study, the arrhythmia-abolishing effect of neither of the drugs is optimal. iPSC-CMs provide a unique platform for testing drugs for CPVT.

## 1. Introduction

Human-induced pluripotent stem cells (hiPSCs) can be generated from patients' somatic cells [1]. The technique makes research of vital organs, such as the heart, available *in vitro*. Primary adult cardiomyocytes (CMs) stop beating *in vitro* after a few hours of culture, but iPSC-derived CMs can be cultured for months with proper beating behavior. The hiPSC-derived CMs carry the same genome as the patient they were generated from. The production of disease-specific hiPSC lines allows disease modeling and drug development *in vitro*.

Catecholaminergic polymorphic ventricular tachycardia (CPVT) is a severe inherited cardiac disease in structurally normal hearts, and it is associated with increased risk of sudden cardiac death [2].The prevalence of CPVT is approximately 1 : 10,000, and the prognosis is bad: mortality rate without treatment is as high as 35% by the age of 30. At the age of 40, 80% of patients have had symptoms. CPVT patients experience arrhythmias during emotional or exercise-induced adrenergic stress. Adrenaline binding into *β*-receptors triggers a signaling cascade via cyclic AMP and protein kinase A, which results in phosphorylation of key Ca^2+^ handling proteins at the membrane of the sarcoplasmic reticulum (SR). In CPVT patients with mutated RYR2, increased Ca^2+^ concentration inside the SR can result in spontaneous Ca^2+^ leakage and in arrhythmias. Exercise stress test is used in the diagnosis of CPVT during which polymorphic ventricular extra systoles or even tachycardia is often seen in the electrocardiograph (ECG).

**Figure 1:**
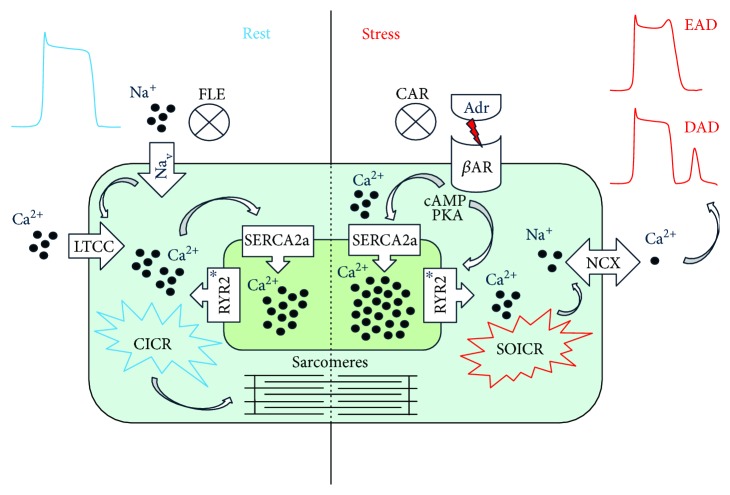
Ca^2+^ overload in the SR during adrenergic stress leads to EADs and DADs causing arrhythmia in CPVT. During rest, depolarization of the CM membrane by inward current of sodium activates LTCCs leading to CICR and contraction. Adrenergic stimulus (Adr) leads to phosphorylation of RYR2 and SERCA2a via second messengers of the *β*-adrenergic pathway (cAMP, PKA) and causes Ca^2+^ overload in the SR. Mutations in RYR2 lead to altered threshold for SOICR and spontaneous Ca^2+^ leak from the SR. Sudden increase in intracellular Ca^2+^ activates NCX and causes membrane depolarization leading to EADs and DADs. Carvedilol (CAR) blocks the beta-adrenergic receptors (*β*AR) inhibiting the phosphorylation of Ca^2+^ handling proteins and decreasing the SR Ca^2+^ load. Flecainide (FLE) inhibits voltage-gated sodium channel (Na_v_) and membrane depolarization leading to the inhibition of LTCC and RYR2 indirectly. CICR = Ca^2+^-induced Ca^2+^ release; SOICR = store overload-induced Ca^2+^ release; NCX = sodium Ca^2+^ exchanger; SERCA2a = sarcoplasmic reticulum Ca^2+^ ATPase; cAMP = cyclic AMP; PKA = protein kinase A. ^∗^Both carvedilol and flecainide have a direct inhibiting effect on RYR2.

Majority of CPVT cases arise from mutations in the gene coding for cardiac ryanodine receptor, *RYR2*. Other genes are also implicated in CPVT, but in far lesser extent, for example, *ANK2*, *CALM1*, *CALM2*, *CASQ2*, *KCNJ2*, *SCN5A*, *TECRL*, and *TRDN* [2]. RYR2 is the largest known ion channel protein located at the membrane of the SR. It releases Ca^2+^ from SR, which acts as an intracellular Ca^2+^ storage in CMs, to the cytosol. In physiologically normal condition, L-type Ca^2+^ channels (LTCCs) at the T-tubules of the sarcolemma are activated by an action potential (AP). LTCCs and RYR2s are located in close proximity to each other creating junctional complexes. The activation of LTCCs creates an inward flow of Ca^2+^ ions into the CM, which in turn activates RYR2s at the adjacent SR. This phenomenon is known as Ca^2+^-induced Ca^2+^ release (CICR) (Figure 1). CPVT causing mutations in RYR2 results in gain of function and altered open probabilities of the channel pore and therefore lowered threshold for store overload-induced Ca^2+^ release (SOICR) [3]. This spontaneous Ca^2+^ leakage can result in sodium Ca^2+^ exchanger (NCX) triggered early and/or delayed afterdepolarizations (EADs/DADs) and arrhythmias in CMs [4].

Beta-blockers are commonly used in the treatment of CPVT. These compounds block the *β*-receptors and thus inhibit the sympathetic stimulation. Carvedilol is a nonspecific, combined *β*1/*β*2/*α*-adrenergic blocker, which has been successfully used in the treatment of chronic heart failure [5]. For patients who do not respond to beta-blocker therapy, flecainide has been investigated as an additional option [6, 7]. During the last few years, it has been intensively investigated by several research groups [8–10]. Flecainide is a class Ic antiarrhythmic agent, a sodium channel blocker, which inhibits inward sodium current via voltage-gated sodium channels (Na_v_1.5). Other therapeutic options for CPVT include implanted cardiac defibrillators and left cardiac sympathetic denervation. However, present therapeutic options do not provide complete protection against potentially lethal arrhythmias, and this is still a clinically relevant problem.

In this study, we evaluate the efficacy of carvedilol and flecainide in abolishing Ca^2+^ abnormalities in CPVT patient-specific hiPSC-derived CMs carrying different mutations in RYR2.

## 2. Methods

### 2.1. Patient-Specific Human iPSC Lines

**Figure 2:**
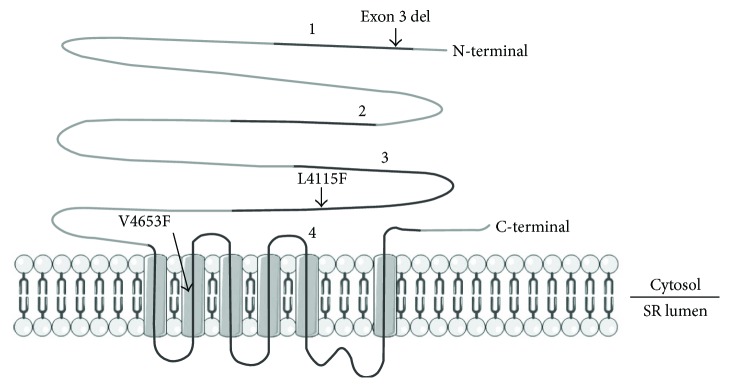
Schematic presentation of RYR2 amino acid sequence and the localization of the studied mutations in it. Mutations in RYR2 are enriched into four clusters (1–4). Modified from [11].

In this study, four CPVT hiPSC lines were used carrying the following *RYR2* mutations: two lines with exon 3 deletion c.168-301_c.273+722del1128 (results pooled), one line with point mutation p.L4115F (c.12343C>T), and another one with point mutation p.V4653F (c.13957G>T). Mutation nomenclature was based on *RYR2* reference sequence NM_001035.2. The location of the mutations in the RYR2 amino acid sequence is illustrated in Figure 2. hiPSC line from a healthy individual was used as a control cell line. Collection of biopsies for generating patient-specific iPSC lines was approved by the ethical committee of Pirkanmaa Hospital District (Aalto-Setälä R08070), and written informed consent was obtained from all the donors. Human iPSC lines were established by sendai viral (CytoTune® iPS reprogramming kit, Thermo Fisher Scientific, Waltham, MA, USA) or retroviral transfection of OCT3/4, SOX2, KLF4, and c-MYC [1]. The characterization of hiPSC lines is described in supplementary data (Supplementary Figures 1 and 2).

### 2.2. Cardiac Differentiation and Dissociation

Differentiation into CMs was carried out by coculturing hiPSCs with murine visceral endoderm-like (END-2) cells (Professor Mummery, Humbrecht Institute, Utrecht, The Netherlands) (supplementary data). hiPSCs formed spontaneously beating clusters after 15 days of coculturing. Beating clusters were cut and isolated with scalpel and dissociated with collagenase A (Roche Diagnostics). Cells were plated on 12 mm round glass coverslips coated with 0.1% gelatin.

### 2.3. Ca^2+^ Imaging

**Figure 3:**
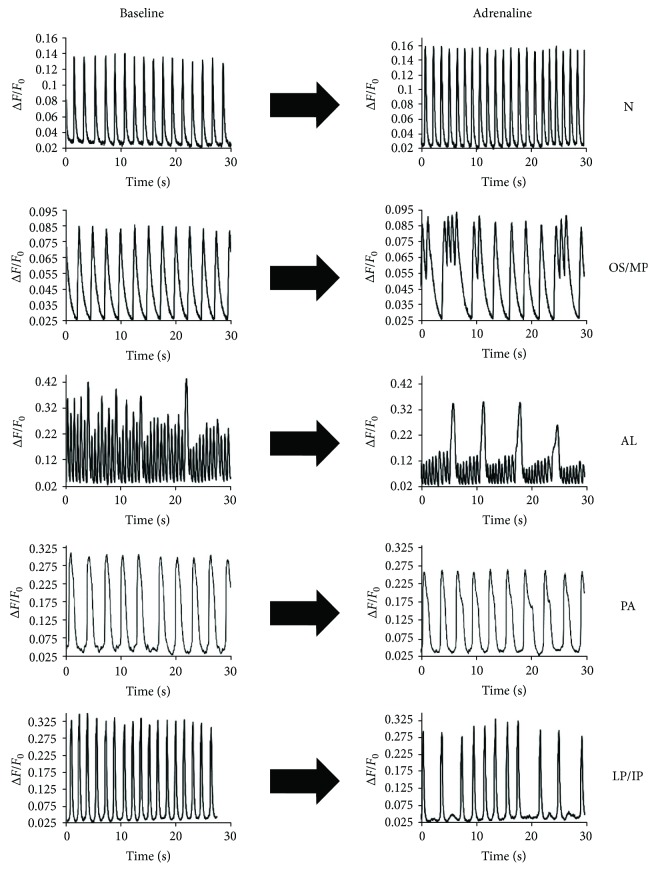
Ca^2+^ transient abnormality types. Normal = N; oscillation = OS; multiple peak = MP; alternans = AL; plateau abnormality = PA; low peaks = LP; and irregular phase = IP. Multiple peaks is two or three consecutive peaks where the amplitude does not decay to the baseline, and oscillation is more than three consecutive kinds of these peaks.

**Figure 4:**
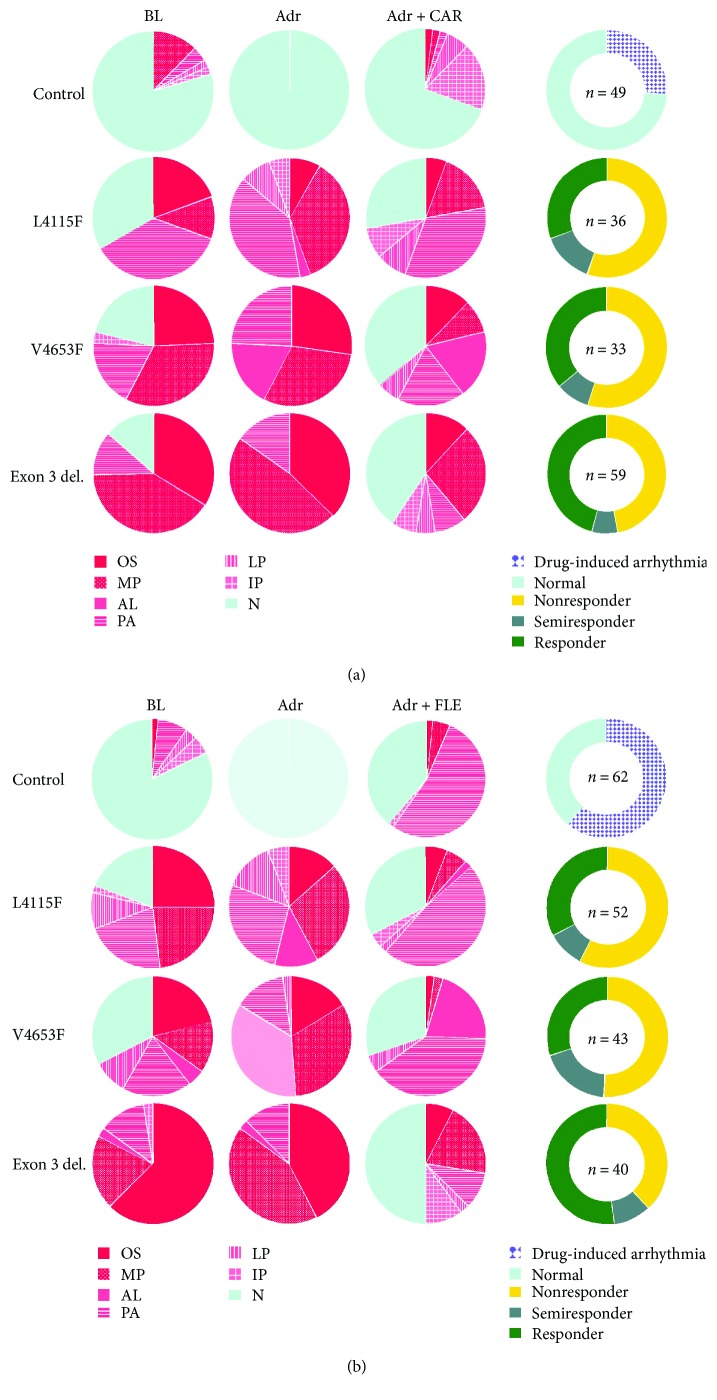
Incidence of different Ca^2+^ abnormality types in control, L4115F, V4653F, and exon 3 deletion CMs under each condition. (a) Carvedilol data and (b) flecainide data. Flecainide caused Ca^2+^ abnormalities in 61% of control cells whereas carvedilol only in 26%. Both drugs were equally effective in CPVT CMs carrying L4115F mutation. V4653F responded the same way to both drugs as L4115F whereas in exon 3 deletion, the efficacy of both drugs was higher. Moreover, in the exon 3 deletion CMs, the incidence of oscillations was much higher than in the others. Donut charts represent the responder groups after drug treatment (adrenaline + drug). BL = baseline; Adr = 1 *μ*M adrenaline; CAR = 0.25 *μ*M carvedilol; FLE = 10 *μ*M flecainide; OS = oscillation; MP = multiple peak; AL = alternans; PA = plateau abnormality; LP = low peak; IP = irregular phase; N = normal; *n* = amount of CMs studied.

**Figure 5:**
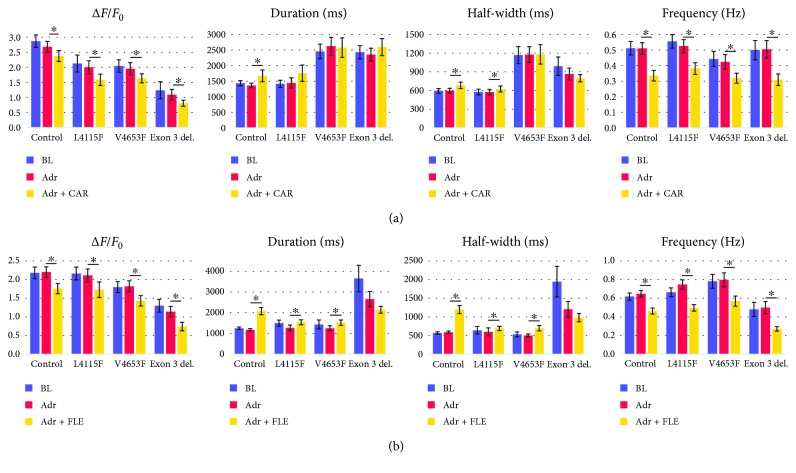
Parameters of the Ca^2+^ traces under each condition. (a) The upper row represents carvedilol data, and (b) the lower row represents flecainide data. Δ*F*/*F*_0_: Ca^2+^ peak amplitude; duration: Ca^2+^ peak duration; half-width: duration at half-maximum amplitude; frequency: the beating frequency of CMs. Data is shown as averages. BL = baseline; Adr = 1 *μ*M adrenaline; CAR = 0.25 *μ*M carvedilol; FLE = 10 *μ*M flecainide. ^∗^Statistical significance (*P* < 0.05). Error bars represent standard error of the mean (SEM).

Ca^2+^ imaging was conducted 4 to 7 days after dissociation, when CMs were 23–78 days old. Cells on a coverslip were loaded with 4 *μ*M Fluo-4 AM (Life Technologies Ltd.) for 30 minutes and deesterified for 10 minutes in perfusate medium: (in mM) 137 NaCl, 5 KCl, 0.44 KH_2_PO_4_, 20 HEPES, 4.2 NaHCO_3_, 5 D-glucose, 2 CaCl_2_, 1.2 MgCl_2_, and 1 Na-pyruvate dissolved in H_2_O. pH of the perfusate medium was adjusted to 7.4 with NaOH. Perfusate was heated to 37°C with an inline heater SH-27B controlled with a TC-324B controller unit (Warner Instruments Inc., CT, USA). A coverslip was attached to a RC-25 imaging chamber (Warner Instruments Inc.) with a silicon-based compound. Ca^2+^ kinetics of spontaneously beating CMs were imaged with an inverted Olympus IX70 microscope using UApo/3400,75NA 20x air objective (Olympus, Tokyo, Japan) and recorded with ANDOR iXon 885 EM-CCD camera (Andor Technology, Belfast, Northern Ireland) using 2 × 2 binning and synchronized with a Polychrome V light source by a real-time DPS control unit. Live Acquisition software (TILL Photonics, Munich, Germany) was used to control light source and camera during recording. Fluo-4 was excited at 490 nm wavelength, and the emission was recorded through Olympus U-MF2 Alexa 488 band-pass filter cube (ex.470–495, em.525/50 nm). Antiarrhythmic evaluation of studied drugs was assessed by recording Ca^2+^ transients at baseline (perfusate only), 3 min after 1 *μ*M adrenaline incubation and if recorded transients exhibited Ca^2+^ abnormalities (Figures 3 and 4), 5 min after 0.25 *μ*M carvedilol (Sigma) or 10 *μ*M flecainide (Sigma) perfusion at the presence of 1 *μ*M adrenaline. All the recordings were 30 sec long with a sampling rate of 20 ms.

### 2.4. Data Analysis and Statistics

For Ca^2+^ imaging analysis, beating single cells or small clusters were selected as regions of interests (ROI) and background signal was subtracted using the Live Acquisition software. Recorded Ca^2+^ traces were further analyzed with Clampfit software version 10.5 (Molecular Devices, USA). The following parameters of the Ca^2+^ traces were extracted: Δ*F*/*F*_0_ (where Δ*F* = *F* − *F*_0_ and *F*_0_ is background subtracted), Ca^2+^ transient duration in milliseconds, half-width (Ca^2+^ transient duration measured at 50% of amplitude) in milliseconds, and frequency in hertz. Statistical analysis was carried out with SPSS software version 23 (SPSS, Chicago, IL, USA). Comparison within cell lines (before and after drug administration) was performed with nonparametric Wilcoxon and between cell lines (cell line to cell line comparison) with nonparametric Mann–Whitney *U* tests with Dunn-Bonferroni post hoc. *P* < 0.05 was considered statistically significant.

Ca^2+^ transients were categorized into seven groups: oscillation (OS), multiple peaks (MP), alternans (AL), plateau abnormality (PA), low peaks (LP), irregular phase (IP), and normal (N) (Figure 3). Ca^2+^ transients were categorized with the following criteria: oscillation, if there were more than three peaks in the same event; multiple peaks, if there were two or three peaks in the same event; alternans, if there were consistent alternating pattern in the amplitude of six or more consecutive peaks in the trace; plateau abnormality, if there was a significant disturbance in the rise or decay phase of a peak (excluding multiple peaks); low peaks, if a single peak amplitude was 10–90% of the amplitude of a normal peak in the trace; irregular phase, if the phase was irregular in the absence of low peaks; and normal, if the trace did not include any of the aforementioned abnormalities. CPVT phenotype of the recorded cells was confirmed by the detection of adrenaline-induced Ca^2+^ abnormalities. Only cells, which exhibited arrhythmias after adrenergic stimulation, were exposed to the drugs. Antiarrhythmic evaluation of studied drugs was assessed after drug and adrenaline incubation phases. If all the Ca^2+^ abnormalities were abolished, recorded trace was categorized into responder (Supplementary Figures 3 and 4). If 50% or more of the abnormalities seen in the adrenaline phase were abolished, trace was categorized into semiresponder. The rest of the traces were categorized into nonresponders. Control cells were exposed to the drugs if the Ca^2+^ transients were normal after adrenergic stimulation (healthy phenotype). Proarrhythmic risk evaluation of the studied drugs in control cells was assessed after administration of either drugs with adrenaline and categorizing responses into two groups: normal, which were completely normal, and drug-induced arrhythmia if one or more arrhythmic events were present in the trace (Supplementary Figures 3 and 4).

## 3. Results

### 3.1. Adrenaline Induced Arrhythmias in CPVT CMs but Abolished Them in Control Cells

All CPVT CMs exhibited arrhythmias after three minutes of *β*-adrenergic stress (1 *μ*M adrenaline), although 25% of L4115F, 28% of V4653F, and 8% of CMs carrying exon 3 deletion were normal at the baseline (Figure 4). All control CMs were normal after three minutes of *β*-adrenergic stimulus, although 19% of the cells showed arrhythmias in the baseline (Figure 4). 1 *μ*M adrenaline abolished arrhythmias in control CMs but induced arrhythmias in CPVT CMs (Figure 4). In CPVT CMs after three minutes of adrenaline incubation, Δ*F*/*F*_0_ was significantly (*P* < 0.05) lower in the oscillation (OS), multiple peaks (MP), and plateau (PA) abnormal transient categories than in the normal (N) transients (Supplementary Figure 5A). In addition, transient duration was significantly (*P* < 0.05) higher in the OS and MP than in the N in the presence of adrenaline (Supplementary Figure 5B). In these two visually most severe abnormality types (OS and MP), the additional arrhythmic events were the reason for prolonged Ca^2+^ peak duration.

### 3.2. Carvedilol and Flecainide Were Equally Effective in CPVT Cells

Both carvedilol and flecainide were equally effective in treating arrhythmias in CPVT CMs. Carvedilol abolished Ca^2+^ abnormalities in 31% of L4115F, 36% of V4653F, and 46% of exon 3 deletion and flecainide 33%, 30%, and 52%, respectively (Figure 4). However, flecainide was superior abolishing AL type of abnormalities compared to carvedilol (Table 1). Both drugs lowered Δ*F*/*F*_0_ of all CMs significantly (*P* < 0.05): carvedilol 12% in control, 21% in L4115F, 16% in V4653F, and 26% in exon 3 deletion and flecainide 20%, 18%, 21%, and 35%, respectively (Figure 5). The intracellular Ca^2+^ was the lowest in CPVT CMs carrying exon 3 deletion, and the antiarrhythmic effect of the drugs was the best in that CPVT cell population. Both drugs also lowered the beating frequency of all CMs significantly (*P* < 0.05): carvedilol 33% in control, 27% in L4115F, 24% in V4653F, and 39% in exon 3 deletion and flecainide 29%, 33%, 29%, and 46%, respectively. CPVT CMs exhibited 25% OS, 37% MP, 11% AL, 21% PA, 4% LP, and 2% IP type of Ca^2+^ abnormalities at the baseline (Table 1). Both drugs abolished most types of arrhythmias: carvedilol abolished 27% OS, 63% MP, 0% AL, 19% PA, 33% LP, and 100% IP and flecainide 32% OS, 51% MP, 27% AL, 20% PA, 57% LP, and 67% IP type of abnormalities.

### 3.3. Mutation-Specific Characteristics

CPVT CMs carrying exon 3 deletion had the highest incidence of OS (39%) and MP (46%) abnormalities compared to other patient-specific CMs after adrenaline exposure (Figure 4). CPVT CMs carrying V4653F mutation had the highest incidence of AL (28%) and CMs with L4115F mutation PA (32%), LP (10%), and IP (6%) abnormalities. The incidence of different abnormal Ca^2+^ transients varied in CMs carrying different mutations. CMs carrying the *RYR2* exon 3 deletion were clearly different compared to the others.

The incidence of PA abnormalities in different *RYR2* mutant CMs treated with flecainide was 10% in exon 3 deletion, 48% in L4115F, and 40% in V4653F and surprisingly also 53% in control (Figure 4(b)). Furthermore, both carvedilol and flecainide were the most effective in abolishing arrhythmias in CMs carrying *RYR2* exon 3 deletion. In addition, carvedilol effect on CMs with exon 3 deletion was dependent of the value of Δ*F*/*F*_0,_ which is a measurement of [Ca^2+^]*_i_*. Responders in this cell population had significantly (*P* < 0.05) higher intracellular Ca^2+^ at baseline and after adrenaline than nonresponders (Supplementary Figure 6A). Overall, exon 3 deletion had the lowest Δ*F*/*F*_0_ values on average throughout the experiments (Figure 5).

### 3.4. Flecainide Caused More Arrhythmias in Control CMs Than Carvedilol

Flecainide caused arrhythmias in 61% and carvedilol in 26% of control cells, which had normal regular transients during adrenaline exposure (Figure 4). Flecainide caused mostly PA abnormalities (87% of arrhythmias), whereas carvedilol caused mostly IP abnormalities (69% of arrhythmias) (Figure 4). The duration and half-width of Ca^2+^ transients were greatly increased in control CMs after flecainide compared to carvedilol (Figure 5). Moreover, in control CMs, flecainide lowered the intracellular Ca^2+^ of nearly double the amount of carvedilol (Figure 5). When comparing normal cells and drug-induced arrhythmias in the control group at the baseline and after adrenaline exposure, CMs with flecainide-induced arrhythmias, duration, and half-width were increased (Supplementary Figures 6C and 6D). At the baseline, the difference in frequency was just bordering statistical significance but reached significance after adrenaline exposure. CMs with drug-induced arrhythmias had also slower beating frequency than normal cells (Supplementary Figure 6E). After flecainide treatment, these differences were even more pronounced. At the baseline and after adrenaline exposure, the control CMs in which carvedilol caused arrhythmias had higher beating frequency but the difference was just bordering the threshold for statistical significance (Supplementary Figure 6B).

## 4. Discussion

In this study, we wanted to mimic the CPVT disease *in vitro* by exposing the hiPSC-derived CMs with adrenaline. CMs generated from different patients carrying different mutations in RYR2 showed different types of calcium abnormalities. Beta-blocker carvedilol and class Ic antiarrhythmic agent flecainide were tested in abolishing these arrhythmias. The patients in the clinic experience arrhythmias under adrenergic stress, and earlier study has shown the hiPSC CMs to provide indicative information of the clinical outcome in drug study [11]. Carvedilol and flecainide were equally effective abolishing Ca^2+^ abnormalities in CPVT CMs, but flecainide was superior abolishing AL abnormalities. Flecainide also caused arrhythmias in 61% of control CMs whereas carvedilol only in 26% of control CMs. CMs with *RYR2* exon 3 deletion were the most responsive to both drug therapies. Furthermore, both drugs lowered the intracellular Ca^2+^ (Δ*F*/*F*_0_) and beating frequency of all CMs, but there were mutation-specific differences in Ca^2+^ abnormality profiles.

### 4.1. Flecainide

Flecainide is thought to have three modes of action. First, as a class Ic antiarrhythmic agent, it is a strong sodium channel blocker. During the fast rise of AP phase 0, depolarization of CM cell membrane takes place and leads to activation of LTCCs and RYR2. Therefore, inhibition of I_Na_ leads indirectly to inhibition of RYR2 action and therefore reduces spontaneous Ca^2+^ leakage from the SR. Second, the ability to block I_Na_ increases the threshold for EAD- and DAD-triggered APs in CMs. The third one is debated, but it is thought that flecainide also has a direct blocking effect on RYR2. Flecainide has already been showing potential in the treatment of CPVT in the clinics [6, 7]. Thus, flecainide was chosen to be tested with our CPVT cell model *in vitro.* In this study, flecainide decreased Δ*F*/*F*_0_ in all CMs but we cannot say if the effect seen was because of direct inhibition of RYR2 or not. The temporal resolution of the recordings in this study did not allow reliable analysis of the rise time of the Ca^2+^ transients, which could have given indication of the effect of I_Na_ block therefore allowing comparison of that between each condition.

The debate about whether flecainide has a direct effect on RYR2 has been recently reviewed [12]. Flecainide effect on RYR2 could be explained by its effect on some of the several RYR2 binding regulators. This would explain why Bannister and coworkers did not see flecainide effect on RYR2 in their permeabilized mouse myocytes or single-channel RYR2 models [8]. Moreover, CMs of mouse origin have a higher beating frequency than those of human origin and flecainide has beat rate-dependent sodium channel-blocking effect; thus, it will probably dominate in mouse models [10]. Contrary to these reports, Watanabe and coworkers and Hilliard and coworkers found direct action of flecainide on RYR2 in isolated myocytes from *CASQ2* knockout mice [7, 9]. However, based on our findings, we cannot exclude mutation-specific effects of flecainide in different CPVT CMs. Opposing views were observed by Liu and coworkers with R4496C and Bannister and coworkers with N4104K mutations in RYR2. Supporting views were studied in wild-type RYR2.

In this current study with control cells, flecainide caused PA abnormalities (Figure 3) with prolongation of plateau phase of Ca_2+_ transients resulting in longer duration and half-width (Figure 5). This is probably a consequence of the ability of flecainide to block I_Kr_/hERG [13]: decreased repolarization rate of CM membrane leads to prolongation in APD. Prolonged APD results in longer open state of LTCCs and NCX and longer Ca^2+^ transient. Furthermore, the control cells, in which flecainide caused arrhythmias, had longer duration and half-width of their Ca^2+^ transients and slower beating frequency after adrenaline exposure than the cells, which were normal (Supplementary Figures 6C–6E). These results go along with the known beat rate dependency of flecainide's sodium channel-blocking ability [14]. 10 *μ*M flecainide blocks 80% and 1 *μ*M only 20% of wild-type hERG current in a HEK293 cell model [13]. Adjusting the flecainide concentration more towards the therapeutic serum levels (0.5–2.4 *μ*M) would probably diminish the percentage of drug-induced arrhythmia in our current study. The majority of flecainide-induced PA abnormalities were seen in the decay phase of Ca^2+^ peaks, and therefore, the prolonged repolarization caused by hERG block would explain these. However, besides the hERG-blocking effect of flecainide, these abnormalities could be also due to reduced intercellular coupling in small cell aggregates due to the sodium channel-blocking effect of flecainide.

The proarrhythmic risk of flecainide has been investigated in the clinical Cardiac Arrhythmia Suppression Trial (CAST) in the mid-90s. In the CAST, patients treated with encainide or flecainide had higher mortality rates after myocardial infarction compared to placebo group [15]. However, another more recent clinical study reported successful treatment of CPVT patients without worsening of exercise-induced ventricular arrhythmias [6]. Class Ic antiarrhythmic agents are known to be associated with proarrhythmia and should be used with caution. Flecainide is contraindicated in structurally abnormal hearts because it slows conduction velocity in myocardium and possibly facilitates reentry. Flecainide is typically used to treat tachycardias, for example, as a rhythm control drug in paroxysmal atrial fibrillation if no other cardiac diseases are present.

For this study, flecainide concentration was selected based on the literature. In the recent study, Preininger and coworkers showed that 10 *μ*M flecainide reduced spontaneous Ca^2+^ waves and Ca^2+^ spark abnormalities by lowering Δ*F*/*F*_0_ of these events in CPVT-specific iPSC-derived CMs carrying L3741P mutation in RYR2 [16]. They compared the effect to *β*-blocker nadolol (10 *μ*M) and found flecainide to be superior. However, they did not see abnormalities in Ca^2+^ activity after flecainide treatment in their control cells. Itzhaki and coworkers tested 10 *μ*M flecainide on iPSC CMs carrying RYR2 M4109R mutation, and it abolished all DADs during patch-clamp recording [17]. Maizels and coworkers [18] studied 6 *μ*M flecainide in CPVT hiPSC CMs carrying mutation in CASQ2. In their study, flecainide abolished 57% of Ca^2+^ abnormalities in CPVT CMs and decreased SOICR incidence by 15%. In our study, flecainide abolished 38% of Ca^2+^ abnormalities in all CPVT CMs, which is less compared to others.

### 4.2. Carvedilol

Beta-blockers can be specific or nonspecific depending of their binding to *β*- and *α*-receptors. Binding to not only *β*-receptors but also *α*-receptors gives a beta-blocker, in addition to its negative inotropic effects on myocardium, a dilative effect on smooth muscle cells of blood vessels therefore reducing also blood pressure. Carvedilol is a versatile nonspecific *β*1/*β*2/*α*-adrenergic blocker used in the treatment of heart failure [5]. Besides carvedilol, nadolol is another common nonselective *β*1/*β*2-blocker, which is used in treatment of CPVT. Adrenergic stress leads to phosphorylation of key Ca^2+^ handling proteins, including RYR2, via the *β*-adrenergic signaling pathway. Efficacy of beta-blockers in the treatment of arrhythmias in CPVT is based on their capability to block this pathway. In addition to its beta-blocking ability, carvedilol and its derivatives have also been shown to suppress SOICR in HEK293 cell model by directly blocking the RYR2 [19, 20]. Therefore, carvedilol has a double action on CM Ca^2+^ handling: via *β*-adrenergic signaling and RYR2. Therefore, carvedilol could have potential in the treatment of CPVT, and thus, it was tested with our CPVT cell model in this study. Also, in the previously mentioned study by Maizels and coworkers, 0.3 *μ*M carvedilol abolished 50% of Ca^2+^ abnormalities in CPVT CMs [18]. Moreover, they reported that carvedilol decreased SOICR incidence by 37%.

In this study, carvedilol was the most effective in CPVT CMs carrying *RYR2* exon 3 deletion. Interestingly, even though these CPVT CMs had lower Δ*F*/*F*_0_ compared to other mutations, the responders in this group had higher Δ*F*/*F*_0_ than the nonresponders (Supplementary Figure 6A). Higher Δ*F*/*F*_0_ was favorable considering responsiveness to the carvedilol therapy in exon 3 deletion CPVT CMs but not in other mutations. Overall, carvedilol decreased Δ*F*/*F*_0_ in all cell lines but, again, we cannot say if the effect seen was because of direct inhibition of RYR2 or not. In control CMs, carvedilol caused arrhythmias in cells, which were beating faster initially, although this finding was not statistically significant (Supplementary Figure 6B). This suggests that in control CMs, in which the spontaneous beating rate is greatly lowered by carvedilol, the irregular beating phase will most probably occur since most of the Ca^2+^ abnormalities caused by carvedilol were IP type.

In this study, we used 1 *μ*M adrenaline concentration, which is relatively high. It is probable that the *β*-receptors were desensitized through the *β*-arrestin-mediated pathway during 3 min administration. Consequently, the *β*-receptors are internalized by endocytosis decreasing the amount of available receptors to the drug molecules. It is possible that due to this reason, we did not see significant increase in beating frequency or changes in duration of calcium transients of the CMs. The effect of adrenaline is rapid and takes place after few seconds of administration *in vitro*. Continuous recording would be ideal, but the phototoxicity of the fluorescent imaging and the adrenaline protocol did not allow a time frame to capture the wanted effect. The main idea was to challenge the cells to mimic the adrenergic stress condition in CPVT. However, if majority of the *β*-receptors were desensitized and internalized, the direct effect of carvedilol on RYR2 could explain the antiarrhythmic efficacy. Carvedilol concentration was selected based on preliminary tests. Concentrations between 0.1 and 1 *μ*M were tested, and higher concentrations stopped the beating completely in majority of the cells.

### 4.3. L4115F and V4653F

Point mutations in RYR2 cause gain of function and spontaneous Ca^2+^ leakage through the channel leading to altered Ca^2+^ handling in CMs. The L4115F and V4653F mutations in RYR2 are located in the mutation clusters 3 and 4, more precisely in the central and channel domains of the protein [21]. Consistent with a previous study, the CPVT cell lines carrying these two mutations were more similar to each other and closer to control cell line compared to the cell line carrying exon 3 deletion [11].

The L4115F CPVT CMs had most PA-, LP-, and IP-type Ca^2+^ abnormalities. They also had a wider spectrum of different Ca^2+^ abnormalities after adrenaline exposure than the others. LP- and IP-type abnormalities could easily be seen arising from DADs (Figure 3). PA abnormalities resemble more EADs, which at the plateau or repolarization phase cause prolongation of Ca^2+^ transients. LP and IP abnormalities instead create small upward fluctuations during diastole, in the baseline of Ca^2+^ transients. In the study by Itzhaki and coworkers with a M4109R RYR2 mutant iPSC-derived CMs, DADs in the phase 4 of APs were the most prevalent arrhythmias seen in patch-clamp recordings [17]. The two aforementioned mutations (L4115F and M4109R) are located close to each other in the RYR2 amino acid sequence, and abnormal transients caused by them are very similar. Even though the studies utilized two different methods, the mutations may have similar arrhythmogenic mechanism creating DADs caused by lowered threshold for SOICR.

A special feature of V4653F CPVT CMs was that they exhibited alternans (AL) abnormalities, which were truly extreme, almost caffeine-like induced eruptions of Ca^2+^ from the SR. Those could be a consequence of altered Ca^2+^ sensitivity of the RYR2 channel opening. Tryptophan being an aromatic amino acid, and not the smallest in size either, could easily be seen creating disruption in tightly packed central and channel domains of the RYR2 altering its function. However, it is hard to say without detailed protein modeling what kind of structural aberrations it could cause in the RYR2 exactly. One noticeable difference in the efficacy of the two studied drugs was the effect of flecainide, and, more precisely, the inefficacy of carvedilol therapy on these AL-type abnormalities. Besides SR Ca^2+^ leak, it is also possible that influx of calcium contributes to this kind of abnormal transients. Therefore, this could explain the partial inhibition of the abnormalities by both of the drugs, since even though they have an effect on the SR calcium leak, they might not be able to affect the Ca^2+^ influx.

### 4.4. Exon 3 Deletion

The exon 3 deletion in RYR2 leads to a severe clinical phenotype of CPVT [22]. The patient carrying the exon 3 deletion had the most polymorphic ventricular complexes at rest during ECG compared to patients carrying L4115F and V4653F mutations [11]. At the protein level, exon 3 deletion causes a structural defect of the cytosolic part of the RYR2 in the N-terminal domain affecting domain-domain interactions inside the protein [21, 22]. Moreover, the ability of RYR2 to rescue itself from a complete unfolding, as a consequence of the exon 3 deletion, via a *β*-strand switch of a flexible loop region renders it more thermally stable [22]. Compared to the gain of function point mutations in RYR2, the Ca^2+^ leak-causing mechanism of exon 3 deletion seems to be much more complicated. It is thought, even though the structural changes were caused by exon 3 deletion, that the allosteric effects to the channel domain would explain the disease-causing malfunction of the Ca^2+^ gating [2]. In addition, the conformational changes, which occur because of the exon 3 deletion, may result in missing or hidden binding sites for RYR2 regulators or coactivators. The aforementioned fundamental differences between the exon 3 deletion and the two studied point mutations probably explain the higher incidence of the more severe OS- and MP-type abnormalities in the CPVT CMs carrying exon 3 deletion. The finding supports another study, which found the exon 3 deletion of RYR2 to be resulted in abnormal termination of Ca^2+^ release through the channel [23]. In addition, OS- and M-type abnormalities had significantly higher Ca^2+^ peak duration and lower Δ*F*/*F*_0_.

Carvedilol and flecainide were equally effective in CPVT cell lines. The antiarrhythmic mechanism of these drugs is based on inhibition of abnormal SR calcium leak and stabilization of [Ca^2+^]*_i_* and therefore suppression of changes in AP which could generate EADs/DADs. Both carvedilol and flecainide were the most effective on the CMs carrying RYR2 exon 3 deletion. Interestingly, the exon 3 deletion is also associated with a severe clinical phenotype [24]. The abnormalities caused by the deletion, mostly OS and MP, were abolished rather well by both drugs. This indicates that the drugs being effective against the OS- and MP-type abnormalities could explain the resulting benefit achieved in the CMS with RYR2 exon 3 deletion.

### 4.5. Limitations

In this study, we did not differentiate between CM subtypes (atrial, nodal, or ventricular). In addition, iPSC-derived CMs are considered to be functionally immature and methods to increase their maturity are intensively investigated in many research groups. Furthermore, it is still unclear how well the calcium abnormalities observed in the iPSC CMs mimic the disease mechanism and whether they predict the treatment efficacy of all drugs in CPVT patients.

## 5. Conclusions

Carvedilol and flecainide were equally effective in treating arrhythmias in CPVT-specific iPSC-derived CMs in this study. However, the proarrhythmic risk of flecainide should be recognized as it induced arrhythmias in control cells. Even though the CPVT CMs carrying exon 3 deletion had the most severe Ca^2+^ abnormalities, it had the best response to the drug therapies. Both of these drugs are used in the clinics for the treatment of CPVT. However, according to this study, the arrhythmia-abolishing effect of these drugs is not optimal at least at these concentrations. iPSC-derived CMs provide a unique platform for testing new potential drugs for CPVT. More detailed studies with combined or simultaneous electrophysiological techniques are needed to fully understand the drug effects and aberrations in excitation contraction coupling leading to arrhythmias in CPVT disease modeling.

## Figures and Tables

**Table 1:** Incidence of each calcium abnormality type in all CPVT CMs after adrenaline stimulation and the percentages of these arrhythmias abolished by either carvedilol or flecainide.

	OS	MP	AL	PA	LP	IP
CPVT CMs	25%	37%	11%	21%	4%	2%
Carvedilol	27%	63%	0%	19%	33%	100%
Flecainide	32%	51%	27%	20%	57%	67%

MP, LP, and IP type of abnormal transients were abolished quite well by both drugs, while flecainide was superior in abolishing AL-type abnormalities. Incidence of LP and IP abnormalities was, however, low in CPVT CMs; therefore, conclusions should be made with caution.

## Supplementary Materials

Supplementary Materials The supplementary material to this manuscript includes detailed description of the materials and methods, additional hiPSC line characterization, and results supporting the data. Supplementary Figure 1: characterization of the UTA.05603.CPVT hiPSC line (exon 3 deletion). Expression of pluripotency markers Nanog, OCT3/4, SOX2, ssea4, tra1-60, and tra1-81 was confirmed in embryoid bodies (EB) by immunocytochemistry. Expression of SOX17, AFP, *α*-cardiac actinin, VEGFR/KDR, SOX1, and Pax6 in EBs was confirmed by PCR. Expression of endogenes Rex1 and *β*-actinin was confirmed by PCR. Inactivation of exogenes OCT3/4, SOX2, KLF4, and c-MYC was confirmed by PCR. Supplementary Figure 2: karyotype analysis of the UTA.05603.CPVT hiPSC line (exon 3 deletion). Karyotype was confirmed normal by PerkinElmer karyolite analysis. Supplementary Figure 3: representative calcium traces from each category before and after the addition of carvedilol. Consecutive traces were recorded from the same cell. In the presence of 1 *μ*M adrenaline, 0.25 *μ*M carvedilol caused calcium abnormalities (IP) in 26% of wild-type cells. Responder CPVT had oscillation (OS) in the baseline and after adrenaline stimulus, but carvedilol abolished all these arrhythmias. Semiresponder still had one multiple-peak event (MP) after carvedilol. In nonresponder, oscillation at baseline and during adrenaline turned into multiple peaks (MP) after carvedilol. Supplementary Figure 4: representative calcium traces from each category before and after the addition of flecainide. Consecutive traces were recorded from the same cell. In the presence of 1 *μ*M adrenaline, 10 *μ*M flecainide caused prolongation of calcium transients (PA) in 53% of wild-type cells. Responder CPVT had oscillation (OS) in the baseline and after adrenaline stimulus, but flecainide abolished all these arrhythmias. In semiresponder CPVT, oscillation at baseline and after adrenaline stimulus turned into irregular beating phase (IP). In nonresponder CPVT, flecainide was not able to abolish alternans (AL) abnormalities. Supplementary Figure 5: comparison of abnormal calcium peak amplitude (ΔF/F_0_) and duration to normal after adrenaline stimulation. (A) Δ*F*/*F*_0_ was significantly (*P* < 0.05) lower in CPVT CMs exhibiting OS, MP, and PA calcium abnormalities compared to normal ones. (B) Duration was significantly (*P* < 0.05) longer in CPVT CMs exhibiting OS and MP (but not PA) calcium abnormalities compared to normal ones. Supplementary Figure 6: comparison of different calcium transient parameters between responders and nonresponders. (A) CMs with exon 3 deletion in which carvedilol abolished all calcium abnormalities had higher Δ*F*/*F*_0_ which indicates [Ca^2+^]*_i_*. Control CMs in which carvedilol caused arrhythmias had higher beating frequency, but the finding was not statistically significant. Control CMs in which flecainide caused arrhythmias had longer calcium peak duration, half-width (C and D), and lower beating frequency (E) CMs. ϯ indicates statistical significance (*P* < 0.05).
