# Supplementary material for: Antiarrhythmic Effects of Carvedilol and Flecainide in Cardiomyocytes Derived from Catecholaminergic Polymorphic Ventricular Tachycardia Patients
Source: Stem Cells Int. 2018 Apr 12;2018:9109503. doi: 10.1155/2018/9109503 (PMC5924967; doi:10.1155/2018/9109503)
Supplement: Supplementary Materials — The supplementary material to this manuscript includes detailed description of the materials and methods, additional hiPSC line characterization, and results supporting the data. Supplementary Figure 1: characterization of the UTA.05603.CPVT hiPSC line (exon 3 deletion). Expression of pluripotency markers Nanog, OCT3/4, SOX2, ssea4, tra1-60, and tra1-81 was confirmed in embryoid bodies (EB) by immunocytochemistry. Expression of SOX17, AFP, α-cardiac actinin, VEGFR/KDR, SOX1, and Pax6 in EBs was confirmed by PCR. Expression of endogenes Rex1 and β-actinin was confirmed by PCR. Inactivation of exogenes OCT3/4, SOX2, KLF4, and c-MYC was confirmed by PCR. Supplementary Figure 2: karyotype analysis of the UTA.05603.CPVT hiPSC line (exon 3 deletion). Karyotype was confirmed normal by PerkinElmer karyolite analysis. Supplementary Figure 3: representative calcium traces from each category before and after the addition of carvedilol. Consecutive traces were recorded from the same cell. In the presence of 1 μM adrenaline, 0.25 μM carvedilol caused calcium abnormalities (IP) in 26% of wild-type cells. Responder CPVT had oscillation (OS) in the baseline and after adrenaline stimulus, but carvedilol abolished all these arrhythmias. Semiresponder still had one multiple-peak event (MP) after carvedilol. In nonresponder, oscillation at baseline and during adrenaline turned into multiple peaks (MP) after carvedilol. Supplementary Figure 4: representative calcium traces from each category before and after the addition of flecainide. Consecutive traces were recorded from the same cell. In the presence of 1 μM adrenaline, 10 μM flecainide caused prolongation of calcium transients (PA) in 53% of wild-type cells. Responder CPVT had oscillation (OS) in the baseline and after adrenaline stimulus, but flecainide abolished all these arrhythmias. In semiresponder CPVT, oscillation at baseline and after adrenaline stimulus turned into irregular beating phase (IP). In nonresponder CPVT, flecain [file 9109503.f1.pdf]

Supplementary material to:

Antiarrhythmic effects of carvedilol and flecainide in cardiomyocytes derived from catecholaminergic polymorphic ventricular tachycardia patients.

Pölönen RP[a], Penttinen K[a], Swan H[b], Aalto-Setälä K[ac].

[a] Faculty of Medicine and Life Sciences and BioMediTech Institute, University of Tampere, Tampere, Finland

[b] Helsinki University Central Hospital, Helsinki, Finland

[c] Heart Center, Tampere University Hospital, Tampere, Finland

M.Sc. Risto-Pekka Pölönen

Arvo Ylpön katu 34

Arvo2 D441

33520 Tampere, Finland

D.*Sc*. (Tech.) Kirsi Penttinen

Arvo Ylpön katu 34

Arvo2 D441

33520 Tampere, Finland

MD PhD Heikki Swan

PO Box 340

00029 Helsinki, Finland

Prof. MD PhD Katriina Aalto-Setälä

Arvo Ylpön katu 34

Arvo2 D437

33520 Tampere, Finland

Corresponding author:

Risto-Pekka Pölönen

Arvo Ylpön katu 34

Arvo2 D441

33520 Tampere, Finland

risto-pekka.polonen@uta.fi

Tel: +35840190415

Human iPS cell culture:

Human iPSCs were cultured on mitomycin-C inactivated mouse embryonic fibroblasts (26,000 cells/cm2, CellSystems Biotechnologie Vertrieb GmbH, Troisdorf, Germany), which act as feeder cells to support the growth of undifferentiated hiPSCs and to maintain their pluripotent state in hiPSC maintenance medium (KO-DMEM (Thermo Fisher Scientific) base, 20% KO-SR (Thermo Fisher Scientific), 1% non-essential amino acids (NEAA, Lonza Group Ltd, Basel, Switzerland), 2mM GlutaMax (Thermo Fisher Scientific), 50 U/ml penicillin/streptomycin (Lonza), 0.1 mM 2-mercaptoethanol (Thermo Fisher Scientific) and 4 ng/ml basic fibroblast growth factor (bFGF, Peprotech, Rocky Hill, NJ, US)). Medium was changed three times per week. The undifferentiated state of hiPSCs was confirmed by morphologic analysis with Nikon Eclipse TS100 phase contrast microscope (Nikon Instruments Europe B.V. Amstelveen, The Netherlands) with Altra-Cell-D-Bundle camera (Olympus Corporation, Tokyo, Japan) and undifferentiated colonies were passaged weekly on new MEFs. MEF feeder cell layers were manually removed by scraping with a pipette tip and treated with 1 mg/ml Collagenase IV (Thermo Fisher Scientific). Remaining attached hiPSC colonies were transferred to a new culture plate. Characterization of hiPSC lines was done as described earlier (1,2)

Cardiac differentiation:

Differentiation into cardiomyocytes was carried out by co-culturing hiPSCs (50,000 cells/cm2) with murine visceral endoderm-like (END-2) cells (prof. Mummery, Humbrecht Institute, Utrecht, The Netherlands)(3). END-2 –cells were mitomycin-C (Sigma Aldrich, St. Louis, MO, USA) inactivated (5 µl/ml, 3h), trypsinized (Lonza) and plated on 12-well-plate (Nunc) 175,000 cells/well in DMEM-F12 (Thermo Fisher Scientific) based medium (7.5% fetal bovine serum (FBS, Biosera, Boussens, France), 1% NEAA (Lonza), 2 mM GlutaMax (Thermo Fisher Scientific) and 50 U/ml penicillin/streptomycin (Lonza)). END-2 –cells were let to attach in 37°C overnight. 0% KO-SR KO-DMEM (Thermo Fisher Scientific) based medium (1% NEAA (Lonza), 2mM GlutaMax (Thermo Fisher Scientific), 50 U/ml penicillin/streptomycin (Lonza) and 0.1mM β-mercaptoethanol (Thermo Fisher Scientific)) was

changed to END-2 cells an hour before plating the hiPSCs. MEFs were removed as described above. hiPSC -colonies were transferred on END-2 –cells ~30 colonies/well with minimal amount of culture medium. Cells were co-cultured in 37°C and 5% CO2. The 0% KO-SR -medium was changed at days 5, 8 and 12. After 15 days of culturing, the medium was changed to 10% KO-SR KO-DMEM (Thermo Fisher Scientific) based medium (10% KO-SR (Thermo Fisher Scientific), 1% NEAA (Lonza), 2mM GlutaMax (Thermo Fisher Scientific), 50 U/ml penicillin/streptomycin (Lonza) and 0.1mM β-mercaptoethanol (Thermo Fisher Scientific)) and changed 3 times a week. Cells were monitored with phase-contrast microscopy (Nikon Eclipse TS100 microscope, Imperx IGV-B1620M-KC000 camera, JAI Camera Control Tool software). hiPSCs formed spontaneously beating clusters after 15 days of co-culturing.

Dissociation of beating cell aggregates:

Beating clusters were cut and isolated with scalpel and dissociated (3). Beating areas were washed in Low-Ca buffer (12ml 1M NaCl, 0.54ml 1M KCl, 0.5ml 1M $MgSO_4$, 0,5ml 1M Na pyruvate, 2ml 1M glucose, 20ml 0,1M taurine and 1ml 1M HEPES (pH adjusted to 6.9 with NaOH)) at room temperature (RT) for 30 minutes and treated with buffer (12ml 1M NaCl, 3µl 1M $CaCl_2$, 0.54ml 1M KCl, 0.5ml 1M $MgSO_4$, 0.5ml 1M Na pyruvate, 2ml 1M glucose, 20ml 0.1M taurine, 1 mg/ml collagenase A (Roche Diagnostics, Basel, Switzerland) and 1ml 1M HEPES (pH adjusted to 6.9 with NaOH)) including collagenase A (Roche Diagnostics) at 37 ˚C for 45 minutes. Cells were incubated in KB medium (3ml 1M K_2HPO_4, 8.5ml 1M KCl, 2mmol/l Na_2ATP, 0.5ml 1M $MgSO_4$, 0.1ml 1M EGTA, 0.5ml 1M Na pyruvate, 2ml 1M glucose, 5ml 0.1M creatine and 20ml 0.1M taurine, pH 7.2) (20µl of 1M glucose was added per 1ml of KB-medium before use) at RT for one hour. Washed beating areas were resuspended in EB-medium (20% FBS (Biosera) in KO-DMEM (Thermo Fisher Scientific), 1% NEAA (Lonza), 2mM GlutaMax (Thermo Fisher Scientific), 50 U/ml penicillin/streptomycin (Lonza)) by pipetting up and down several times against the bottom of the dish.

Referenecs:

(1) Ahola A, Kiviaho AL, Larsson K, Honkanen M, Aalto-Setälä K, Hyttinen J. Video image-based analysis of single human induced pluripotent stem cell derived cardiomyocyte beating dynamics using digital image correlation. Biomedical engineering online 2014;13(1):39.

(2) Kujala K, Paavola J, Lahti A, Larsson K, Pekkanen-Mattila M, Viitasalo M, et al. Cell model of catecholaminergic polymorphic ventricular tachycardia reveals early and delayed afterdepolarizations. PloS one 2012;7(9):e44660.

(3) Mummery C, Ward-van Oostwaard D, Doevendans P, Spijker R, van den Brink S, Hassink R, et al. Differentiation of human embryonic stem cells to cardiomyocytes: role of coculture with visceral endoderm-like cells. Circulation 2003 Jun 03,;107(21):2733-2740.

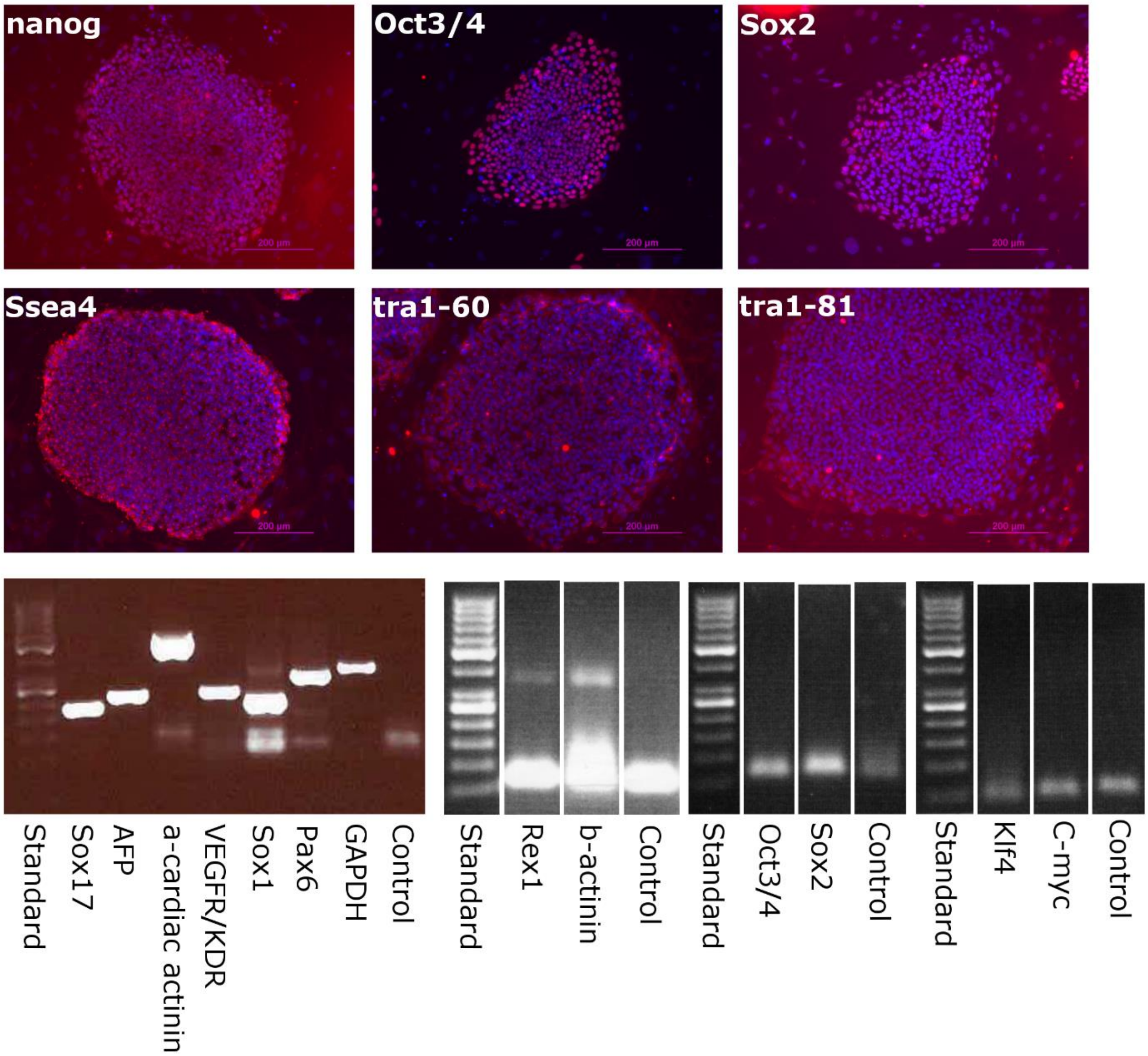

Supplementary figure 1. Characterization of the UTA.05603.CPVT hiPSC line. Expression of pluripotency markers nanog, oct3/4, sox2, ssea4, tra1-60 and tra1-81 was confirmed in embryoid bodies (EB) by immunocytochemistry. Expression of Sox17, AFP, α-cardiac actinin, VEGFR/KDR, Sox1, and Pax6 in EBs was confirmed by PCR. Expression of endogenes rex1 and β-actinin was confirmed by PCR. Inactivation of exogenes oct3/4, sox2, klf4 and c-myc was confirmed by PCR.

Centre for Biotechnology
The Finnish Microarray and Sequencing Centre
Tykistokatu 6 A; 5th floor
20520 Turku
Finland
fmsc@btk.fi

Sample Id:	UTA 05603_1
CSV File Name:	Karyolite 150320_20150320_095608
CSV File Generation Date:	3/20/2015 8:14:00 AM
Ref. Autosomal CV%:	4.61(Target:<6%)
Ref. Autosomal MFI:	320
Ref. Total X Separation:	0.84(Target:>0.6)
Sample CV%:	6.32(Target:<8%)
Threshold:	0.874 - 1.126
Threshold Y:	0.811 - 1.189
BoBsoft Panel Name:	UnknownToBoBsoft
Algorithm Name:	Ratio
Reference Method:	M/F Reference DNA
BoBsoft Analysis Date:	3/23/2015 7:57:45 AM
User name:	Marjo Hakkarainen

Normalized Ratios

Probe	/F	/M	Chromosomal Location
01C	0.98	1.03	
02C	1.00	1.00	
03C	0.99	1.01	
04C	0.97	1.02	
05C	0.95	0.97	
06C	1.00	0.99	
07C	1.09	1.10	
08C	0.93	0.92	
09C	1.04	1.07	
10C	0.98	1.00	
11C	1.00	0.96	
12C	1.00	1.01	
13C	1.00	1.06	
14C	1.01	1.03	
15C	0.94	1.01	
16C	1.00	1.04	
17C	0.98	0.96	
18C	0.96	1.02	
19C	1.06	1.03	
20C	1.00	1.03	
21C	0.96	0.92	
22C	1.04	1.05	
Auto	0.96	0.97	
X	1.03	1.54	
Y	1.02	0.48	

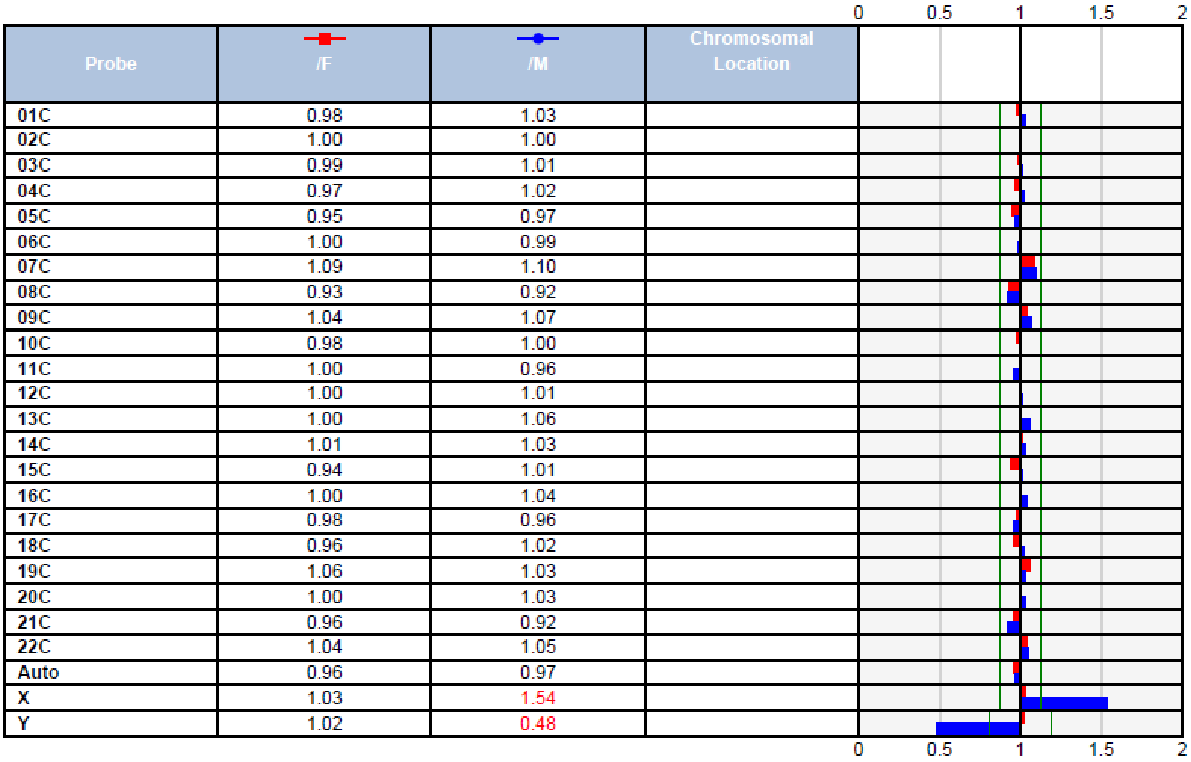

Supplementary figure 2. Karyotype analysis of the UTA.05603.CPVT hiPSC line. Karyotype was confirmed normal by PerkinElmer Karyolite analysis.

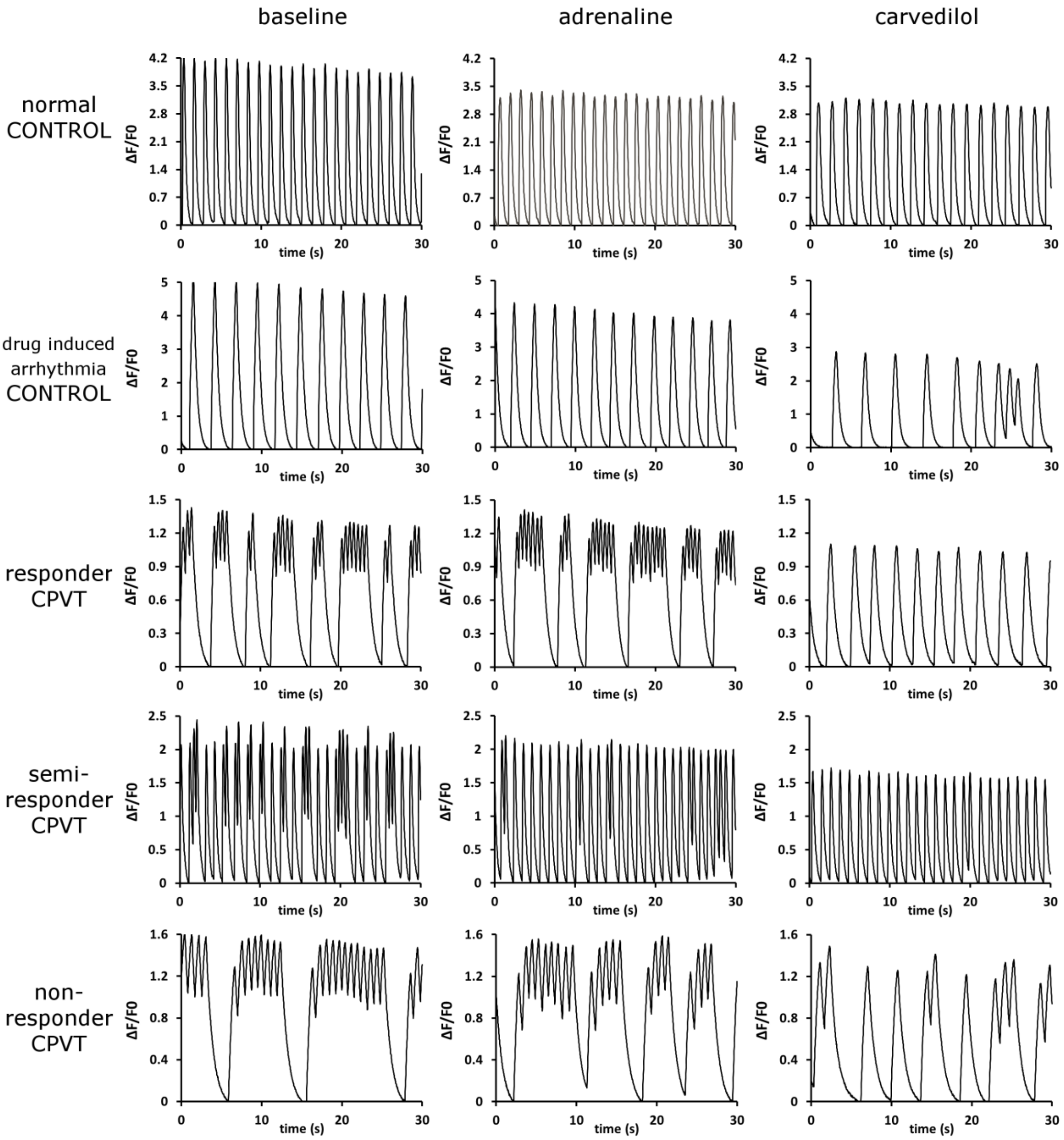

Supplementary figure 3. Representative calcium traces from each category before and after the addition of carvedilol. Consecutive traces were recorded from the same cell. In the presence of 1µM adrenaline, 0.25µM carvedilol caused calcium abnormalities (IP) in 26% of wild type cells. Responder CPVT had oscillation (OS) in the baseline and after adrenaline stimulus, but carvedilol abolished all these arrhythmias. Semi-responder still had one multiple peak event (MP) after carvedilol. In non-responder oscillation at baseline and during adrenaline turned into multiple peaks (MP) after carvedilol.

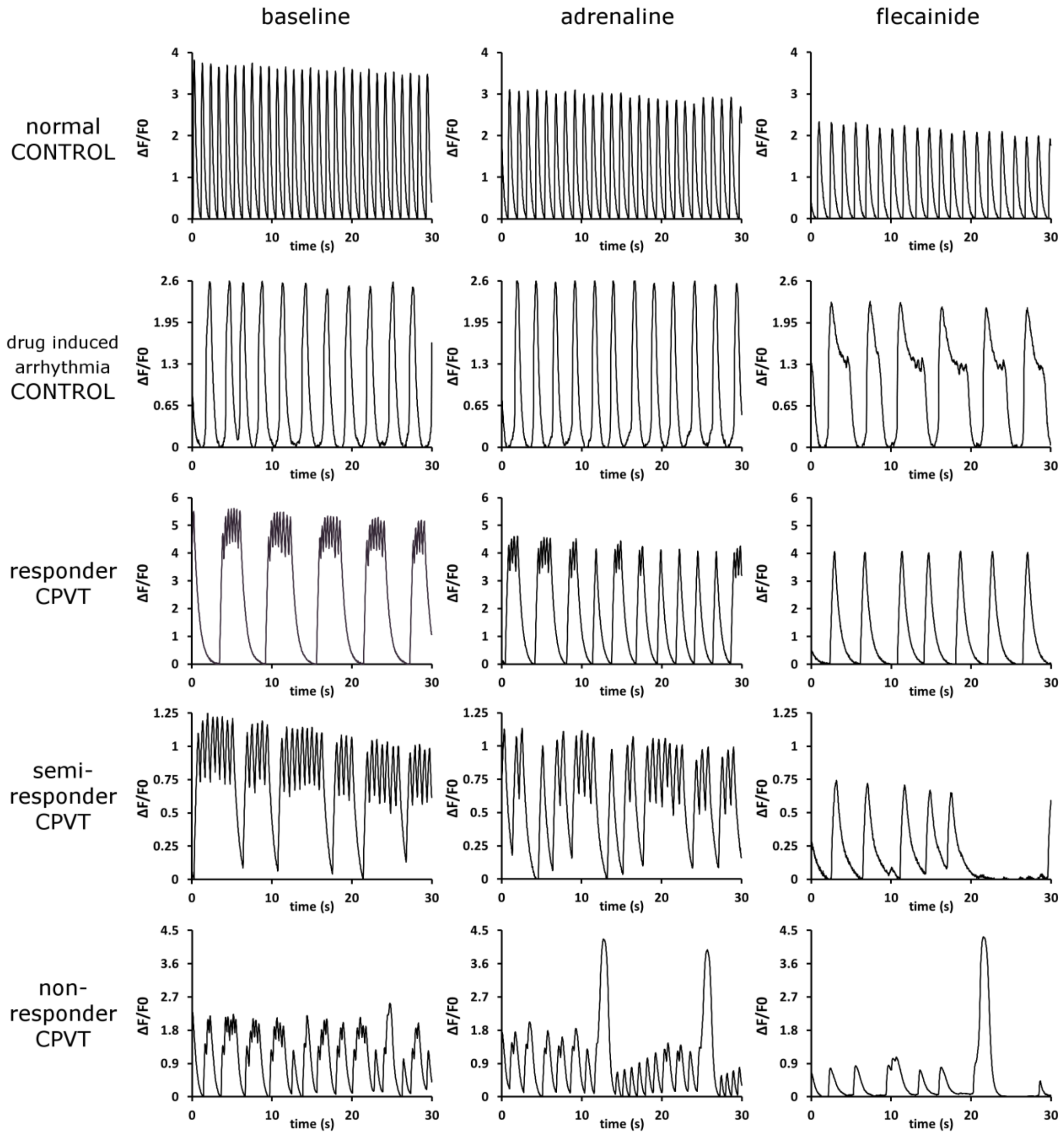

Supplementary figure 4. Representative calcium traces from each category before and after the addition of flecainide. Consecutive traces were recorded from the same cell. In the presence of 1μM adrenaline, 10μM flecainide caused prolongation of calcium transients (PA) in 53% of wild type cells. Responder CPVT had oscillation (OS) in the baseline and after adrenaline stimulus, but flecainide abolished all these arrhythmias. In semi-responder CPVT, oscillation at baseline and after adrenaline stimulus turned into irregular beating phase (IP). In non-responder CPVT, flecainide was not able to abolish alternans (AL) abnormalities.

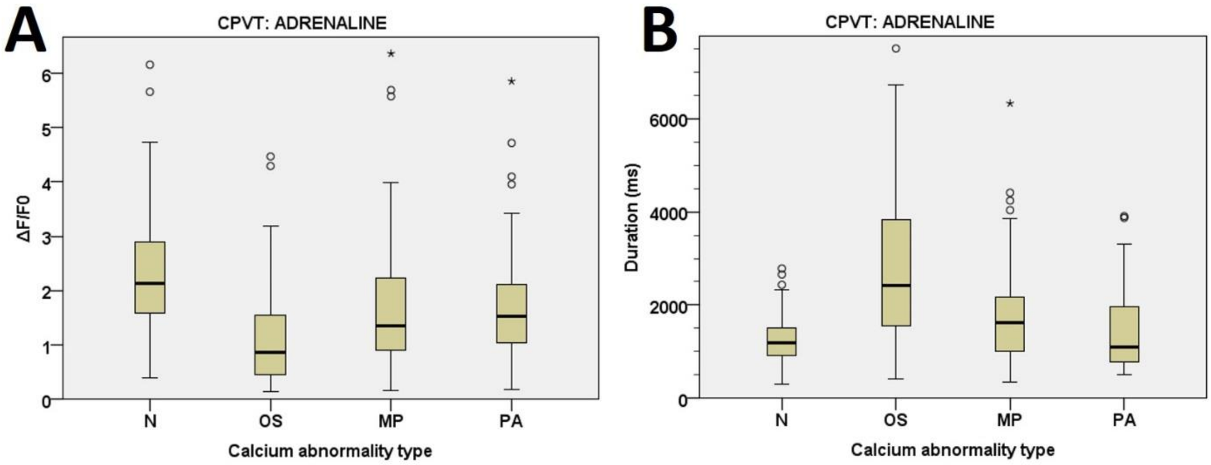

Supplementary figure 5. Comparison of abnormal calcium peak amplitude ($\Delta F/F_0$) and duration to normal after adrenaline stimulation. (A) $\Delta F/F_0$ was significantly ($p<0.05$) lower in CPVT CMs exhibiting OS, MP and PA calcium abnormalities compared to normal ones. (B) Duration was significantly ($p<0.05$) longer in CPVT CMs exhibiting OS and MP (but not PA) calcium abnormalities compared to normal ones.

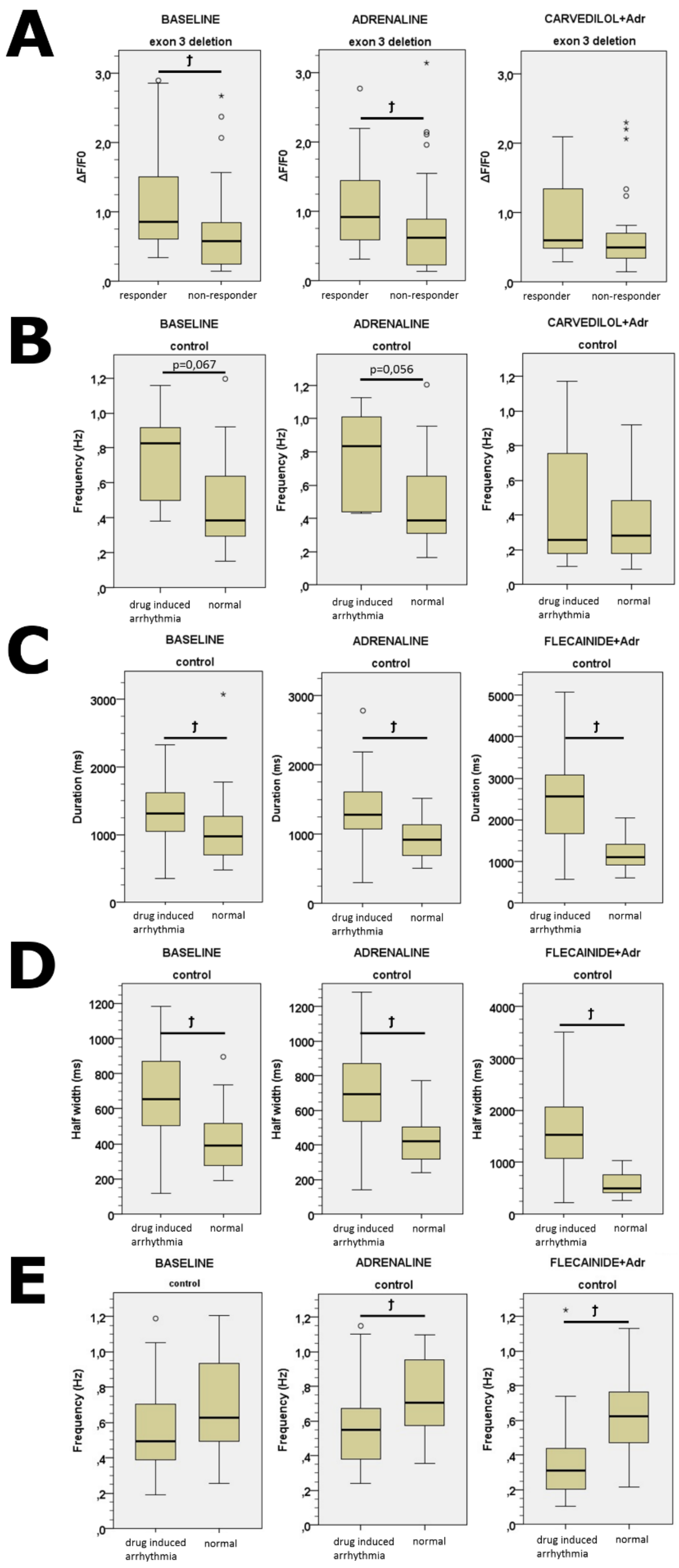

Supplementary figure 6. Comparison of different calcium transient parameters between responders and non-responders. (A) CMs with exon 3 deletion in which carvedilol abolished all calcium abnormalities had higher

$\Delta F/F_0$ which indicates $[Ca^{2+}]_i$. Control CMs in which carvedilol caused arrhythmias had higher beating frequency but the finding was not statistically significant. Control CMs in which flecainide caused arrhythmias had longer calcium peak duration, half width (C and D) and lower beating frequency (E) CMs. ǂ indicates statistical significance ($p<0.05$).
